# Supplementary material for: Multi‐dataset identification of innovative feature genes and molecular mechanisms in keratoconus
Source: J Cell Mol Med. 2024 Sep 19;28(18):e70079. doi: 10.1111/jcmm.70079 (PMC11412914; doi:10.1111/jcmm.70079)
Supplement: Supplementary file 2 — Material S2. [file JCMM-28-e70079-s002.zip › 7.GSEA analysis/1.ARL11/KEGG_PENTOSE_AND_GLUCURONATE_INTERCONVERSIONS.html]

Details for gene set KEGG\_PENTOSE\_AND\_GLUCURONATE\_INTERCONVERSIONS[GSEA]

|  || Dataset | ARL11.group.cls#h\_versus\_l |
| Phenotype | group.cls#h\_versus\_l |
| Upregulated in class | h |
| GeneSet | KEGG\_PENTOSE\_AND\_GLUCURONATE\_INTERCONVERSIONS |
| Enrichment Score (ES) | 0.43013915 |
| Normalized Enrichment Score (NES) | 1.1433965 |
| Nominal p-value | 0.29038855 |
| FDR q-value | 1.0 |
| FWER p-Value | 0.995 |
Table: GSEA Results Summary

  

Fig 1: Enrichment plot: KEGG\_PENTOSE\_AND\_GLUCURONATE\_INTERCONVERSIONS      
 Profile of the Running ES Score & Positions of GeneSet Members on the Rank Ordered List

  

| SYMBOL | TITLE | RANK IN GENE LIST | RANK METRIC SCORE | RUNNING ES | CORE ENRICHMENT || 1 | UGT2B7 | na | 1178 | 0.368 | 0.1026 | Yes |
| 2 | UGT2B15 | na | 2914 | 0.244 | 0.1434 | Yes |
| 3 | UGT2B11 | na | 2923 | 0.244 | 0.2337 | Yes |
| 4 | UGT2B4 | na | 3576 | 0.216 | 0.2952 | Yes |
| 5 | UGT1A8 | na | 3762 | 0.209 | 0.3676 | Yes |
| 6 | UGT1A6 | na | 4803 | 0.172 | 0.4014 | Yes |
| 7 | UGT2B28 | na | 5683 | 0.146 | 0.4301 | Yes |
| 8 | DCXR | na | 8022 | 0.092 | 0.3969 | No |
| 9 | DHDH | na | 9329 | 0.070 | 0.3852 | No |
| 10 | UGT2B10 | na | 9893 | 0.062 | 0.3918 | No |
| 11 | UGT2A3 | na | 13634 | 0.022 | 0.2922 | No |
| 12 | GUSB | na | 16664 | -0.001 | 0.2053 | No |
| 13 | RPE | na | 19229 | -0.019 | 0.1383 | No |
| 14 | CRYL1 | na | 19590 | -0.021 | 0.1357 | No |
| 15 | UGDH | na | 19859 | -0.023 | 0.1365 | No |
| 16 | UGT2A1 | na | 20689 | -0.029 | 0.1233 | No |
| 17 | UGP2 | na | 20718 | -0.029 | 0.1333 | No |
| 18 | AKR1B1 | na | 30604 | -0.208 | -0.0744 | No |
| 19 | XYLB | na | 31684 | -0.247 | -0.0136 | No |
| 20 | RPEL1 | na | 32266 | -0.273 | 0.0709 | No |
Table: GSEA details [plain text format]

  

Fig 2: KEGG\_PENTOSE\_AND\_GLUCURONATE\_INTERCONVERSIONS      
 Blue-Pink O' Gram in the Space of the Analyzed GeneSet

  

Fig 3: KEGG\_PENTOSE\_AND\_GLUCURONATE\_INTERCONVERSIONS: Random ES distribution      
 Gene set null distribution of ES for **KEGG\_PENTOSE\_AND\_GLUCURONATE\_INTERCONVERSIONS**
